# Physical Activity, Climate Change and Health—A Conceptual Model for Planning Public Health Action at the Organizational Level

**DOI:** 10.3390/ijerph19084664

**Published:** 2022-04-12

**Authors:** Sven Schneider, Alexandra von Winning, Fiona Grüger, Stefan Anderer, Robert Hoffner, Lilian Anderson

**Affiliations:** 1 Center for Preventive Medicine and Digital Health Baden-Württemberg, Medical Faculty Mannheim, Heidelberg University, D-68167 Mannheim, Germany; landerso@mail.uni-mannheim.de; 2 Lust auf Besser Leben gGmbH, D-60385 Frankfurt am Main, Germany; alexandra@lustaufbesserleben.de; 3 Department of Education and Qualification, Sports Association of North Baden, D-76131 Karlsruhe, Germany; f.grueger@badischer-sportbund.de; 4 Department Sports and Society, Sports Confederation of Württemberg, D-70372 Stuttgart, Germany; stefan.anderer@wlsb.de; 5 Department Sports Facilities, Sports and Exercise Rooms, Municipal Advice, Sports Confederation of Württemberg, D-70372 Stuttgart, Germany; robert.hoffner@wlsb.de

**Keywords:** physical activity, public health, sport, risk, climate change, population

## Abstract

Climate change is linked to health risks for both professional and amateur athletes. Sports organisations will need to react to these developments. The starting point for this concept paper is a summary of the sport-specific health risks currently under discussion: increasing heatwaves, growing numbers of extreme weather events, rising UV, ozone and allergen levels and the spread of infectious diseases. Based on the current state of research, a conceptual model is developed to reduce these climate-related health risks in sports at organisational level. Given the wide variety of predicted direct and indirect health risks linked to climate change, the “sports, clubs and climate change model” (SC^3^ model) presented here follows a stepwise risk-specific approach using technical, organisational and person-related measures. The SC^3^ model also includes cross-cutting measures that have an overarching effect comprising training, warning systems, coordination and evaluation measures. The SC^3^ model makes it possible to develop prevention plans, both at national level for central associations and at the regional level of local organisations and clubs. It can be applied to typical settings (e.g., training or competition at elite or amateur levels) and target groups (e.g., athletes, spectators, referees and club officials).

## 1. Introduction

Climate change represents what is probably the greatest challenge to face humanity this century [1,2]. In light of the large proportion of people who actively participate in any form of physical activity within the total population of Western industrialised countries, it is surprising that relatively little attention has been given to the significance of climate change for sport [3]. Meanwhile, numerous sports associations and clubs, as well as the athletes belonging to these organisations, are already confronted to varying degrees with the adverse effects of climate change on health, infrastructure and process organisation.

The first section of this concept paper outlines the various ways in which climate change affects health. The second section presents a new intervention model that can be used to deal with climate-related health risks. This “sports, clubs and climate change model” (SC^3^ model) makes it possible to develop prevention plans, both at national level for central associations and at the regional level of local organisations and clubs. It can be applied to individual sports, as well as to typical settings (e.g., training or competition at elite or amateur levels) and target groups (e.g., athletes, spectators, referees and club officials).

## 2. The Problem

### 2.1. Increased Thermal Stress

Temperatures will continue to rise in many areas of the world as a result of climate change. In 2020, the average global temperature was already 1.2 °C above pre-industrial levels. In many regions, 2020 was the warmest year ever recorded [4]. Current forecasts predict that the average temperature will continue to rise steeply until 2100 [5]. At the same time, climate change will also cause a considerable increase in the frequency, severity and duration of short- to medium-term extreme air temperatures in the summer [6]. Higher outdoor temperatures typically cause increased stress on the cardiovascular, respiratory and metabolic systems [7]. Sustained periods of higher temperatures—referred to as heatwaves—result in heat stress, which the body reacts to by acclimatising. During this process, plasma volume increases, perspiration rate quickens, and sweat production kicks in earlier [8]. If the high outdoor temperature is accompanied by high levels of humidity, sweat output and therefore also the body’s ability to regulate its temperature are particularly at risk [8,9]. This can then rapidly escalate to an acute emergency situation [9]. Sports institutions such as clubs, associations, business operators and event organisers are tasked with protecting athletes, referees, staff and spectators from the effects of hyperthermia (heat stroke, collapse, heat exhaustion and heat cramps) during heatwaves [10]. This applies to both outdoor and indoor sports. Sports halls and club houses heat up if the buildings have poor energy efficiency standards. Furthermore, summer drought periods cause playing areas, pitches and fields to dry out. In addition to this, winter sports are experiencing a decline in the number of areas where snow is guaranteed.

### 2.2. Extreme Weather Events

Another consequence of climate change is the acceleration of the water cycle [4]. This results in a net increase in regional annual precipitation levels. In the middle latitudes, increasing precipitation tends to occur in the winter half of the year, while the summer months can be expected to become drier [11]. An acceleration of the water cycle accompanied by advancing global warming has the effect that precipitation in winter increasingly falls as rain instead of snow and that extreme weather such as heavy rainfall is to be expected more frequently in the summer [12]. For sports clubs, more extreme weather events mean an increased risk to athletes, sports grounds and facilities from continuous rainfall, flooding, lightning strikes and falling tree branches [13]. Excessive rainfall can cause flooding and the washing away of synthetic granules at club-owned facilities (e.g., tennis courts, artificial grass pitches), as well as causing penetrating damp and mould infestations in buildings (e.g., sports halls and club houses). In addition to this, dangerous high and low tides pose a threat to water sports.

### 2.3. Increased UV Exposure

In the last century, the ongoing emission of greenhouse gases led to the depletion of the ozone layer in the stratosphere (“ozone holes”), thus causing an increase in low-lying UVB radiation [14]. A rise in the number of stratospheric low ozone events (“mini ozone holes”) in the middle latitudes of the northern hemisphere has also been observed [15]. In addition to this, climate change has also led to an increase in average sunshine duration in recent decades [16]. Overall, these changes tend to result in an acute and chronic increase in UV exposure for athletes. Sport institutions have a responsibility to protect athletes, coaches, animals and—at matches and competitions—referees, staff and spectators from both the acute effects (e.g., UV erythema and sunstroke) and the chronic consequences (e.g., photoaging, cataracts, non-melanocytic skin cancer, malign melanoma) of excess UV exposure [10,17]. It should be noted that the thermal risks described above do not necessarily occur together with the UV-related risks discussed here, e.g., a hot and humid but overcast day in summer vs. a cloudless sunny day in spring.

### 2.4. Increased Air Pollution

Ground-level ozone is an indicator of secondary photochemical pollutants, which are formed from motor traffic emissions (nitrogen oxides and hydrocarbons) when exposed to UV radiation [18]. Adverse ground-level ozone values primarily occur in the summer months and, counter-intuitively, are more common in wooded areas than in urban regions [18]. Since an increase in stable high-pressure weather conditions with high average temperatures and periods of extreme heat is expected in the future, climate models therefore predict an increase in levels of ozone pollution [19]. Ozone causes inflammation in the alveoli, makes the body more prone to infection and—especially during physical exertion—can lead to a deterioration in lung function, coughing, tiredness and reduced performance levels [20]. Advice from the WHO states that ozone levels averaging 100 μg/m^2^ over 8 h are already enough to have an impact on our health. Significant effects can be expected from 160 μg/m^2^, and levels of 240 μg/m^2^ and above can be expected to have a severe influence on health [20].

Climate change induces specific weather conditions (heatwaves and droughts) which can also lead to a significant increase in particulate matter pollution [21]. Furthermore, the combination of heat, ozone and particulate matter results in strong synergistic, additive interactions. Particles smaller than 2.5 μm (PM_2.5_) are especially harmful as these can penetrate the alveoli and lead to a diffusion of pro-inflammatory signalling molecules, atherosclerotic processes and chronic changes to the respiratory tract [22].

### 2.5. Increased Exposure to Allergens

As a result of climate change and the resulting milder weather conditions, seasonal pollen calendars are beginning to start earlier and end later [23,24]. In addition to the length of the pollen season, climate change also affects the concentration and allergenic potential of pollen [25]. It is assumed that the increased concentrations of atmospheric CO_2_ and the sustained rise in temperatures will have a positive effect on biomass production in the form of a “fertilisation effect” [26]. In addition to this, air pollutants are causing the protein composition of plant pollen to change, making the pollen more aggressive. The changes to our climate detected in recent decades are also favourable for the naturalisation of neophytes with allergenic potential (e.g., Ambrosia artemisiifolia) [27]. For many athletes affected by this, allergic symptoms include allergic rhinitis (pollinosis) and asthma. Medical treatment of these complaints can cause additional problems for athletes and sports clubs if drugs are used that are relevant in terms of doping [28].

### 2.6. Spread of Infectious Diseases

In the wake of climate change, there is a discussion regarding the increased spread of vectors (particularly ticks and mosquitoes) and natural reservoirs (e.g., migratory birds). The majority of vectors are ectothermic animals whose living conditions are generally improved by global warming [29].

Mild winters produce favourable conditions for earlier activity and a high density of ticks. Ticks are carriers of Q fever, Rickettsia, Ehrlichia, Lyme disease and tick-borne encephalitis (TBE) [30]. In general, climate change also creates more favourable conditions for the proliferation and survival of other vectors such as the Anopheles mosquito, the sand fly (*Phlebotiminae*) and the Asian tiger mosquito (*Aedes albopictus*). Mosquitoes can carry diseases such as chikungunya fever, dengue, Zika virus and West Nile virus, as well as malaria. At the same time, climate change also affects the flight paths of migratory birds that act as natural reservoirs and which can import new pathogens [31,32]. People participating in outdoor sports are at particular risk from vector-related infections. 

Climate change also compromises water quality and encourages favourable conditions for water-borne infections and poisoning. In fresh water, this can lead to the massive growth of cyanobacteria, creating algal blooms, whilst in brackish water, this can lead to the accumulation of Vibrio bacteria (*Vibrio vulnificus*) [32,33]. Due to their ability to produce toxins, these bacteria pose a risk to water sports enthusiasts in particular. 

Finally, it should also be mentioned that climate change also leads to an increased risk of food-borne infections (especially from *Salmonella*, *Campylobacter jejuni* and *Campylobacter coli*), particularly at sports events where a seamless cooling chain cannot be guaranteed for the food and beverages served.

### 2.7. Sport-Specific Advantages of Climate Change

Finally, the positive effects of climate change on the world of sport also need to be considered. In general, global warming is likely to lead to an increase in average comfort levels over the year [34]. For many types of outdoor sports, this means that the outdoor season can start earlier and finish later [35]. This in turn increases sports hall capacity for competing indoor sports. Extended seasons and more sunny days will increase annual exposure to UV radiation. This can have a preventive effect on symptoms of depression (seasonal affective disorder), boost vitamin D levels and reduce the risk of deficiency-related diseases such as osteoporosis [36]. Sports clubs will face additional costs in the summer for irrigation and air conditioning, countered by reduced heating needs during the winter months. A greater number of snow- and ice-free days will also mean a reduction in costs for winter weather services, clearing playing areas and heating grass pitches.

## 3. The Intervention Model

**Figure 1:**
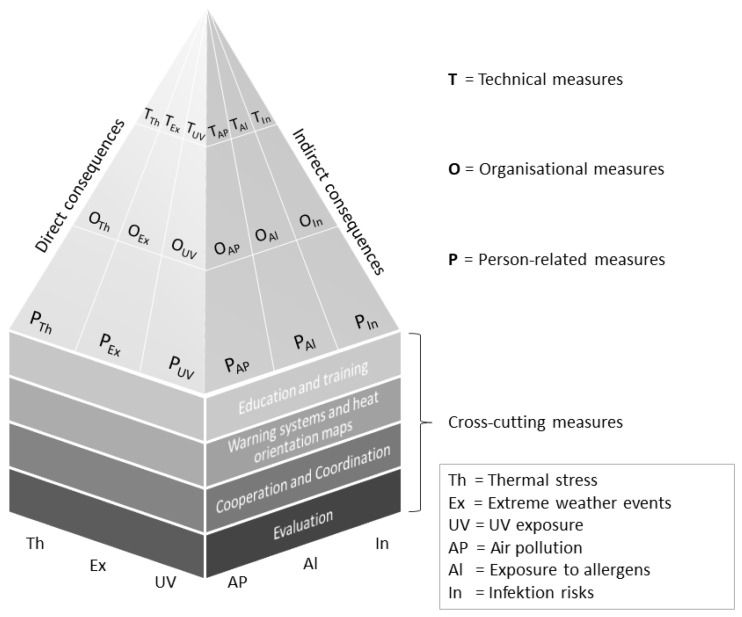
Conceptual model to reduce climate-related health risks in sports at organisational level (“sports, clubs and climate change model”—SC^3^ model).

We aim to develop an intervention model (“sports, clubs and climate change model—SC^3^ model”; Figure 1) to counter the manifold health risks related to climate change. It is compatible with the “TOP principle” commonly used in occupational safety as well as with the concept of “heat health action plans” used in public health [6,37]. According to the “TOP principle”, workplace-specific risks should first be prevented using technical, structural measures (T), i.e., creating and changing local infrastructure. If this is not sufficient, then the “TOP principle” states that additional organisational measures (O) should be implemented. Such measures involve organisational structures and procedures and protect target groups irrespective of their individual behaviour (e.g., altering training times, security audits, additional breaks). In contrast, person-related measures (P) only fulfil their purpose of further reducing any remaining residual risk if the addressees actually make use of what is provided (e.g., handing out water bottles and sun protection products, providing disinfectant dispensers) [38].

Given the wide variety of predicted health risks linked to climate change, we propose a risk-specific TOP approach in each case (Table 1). To this end, we categorised the abovementioned health risks as direct and indirect consequences of climate change. Direct consequences are primarily caused by extreme temperatures and other extreme weather conditions. Indirect consequences are the result of climate-induced changes to our ecosystem (Figure 1) [39]. In addition to this, the “sports, clubs and climate change model” also includes cross-cutting measures that have an overarching effect. The four cross-cutting measures are based around public health concepts, the core components of which comprise training measures, warning systems and coordination and evaluation measures (Table 1) [6,37,39].

### 3.1. Technical Measures

Sports clubs can implement technical structural measures to diminish heat stress. The effects of heat in sports halls can be reduced using modern insulation technology and renovation measures designed to improve energy efficiency. Even simple measures can help, such as the use of light-coloured paint on buildings and modern window glazing. Roof and facade greening complete with weather-resistant vegetation has a positive effect on the microclimate thanks to its insulating, shade and air filtration properties and the associated evaporation processes [37]. Trees can provide shade, protect from erosion and create a cooling effect through evaporation.

It is not only athletes that suffer in the heat but also grass playing fields [3]. This can be countered by transitioning to the use of warm-season grasses and using climate-friendly lawn maintenance practices, such as increasing the mowing height, using an automated underground irrigation system and climate-adapted soil remediation.

People participating in outdoor sports can be protected from extreme weather events such as storms and lightning strikes by providing and designating lightning protection shelters and rooms, as is already done on golf courses [17].

The athletes themselves are also to be protected from UV radiation. In certain outdoor sports disciplines (e.g., athletics), suitable trees or awnings can be used to create natural or artificial shade over at least some areas of the pitch or training ground. In many ball sports, it is not generally possible to create an area of shade over the actual playing area (e.g., football, hockey, tennis) or it would be very expensive (e.g., beach volleyball, beach handball, beach football) [40]. In this case, shade should at least be guaranteed for the athletes during set-up times and breaks, for example, with covered stands and coaching zones. Furthermore, especially at major events, the vast majority of people exposed to UV radiation comprises spectators, coaches, referees and service staff. Protecting all of these people from UV radiation poses a particular challenge.

Localised exposure to ozone, PM_2.5/10_ and allergens can likewise be reduced by implementing technical or structural measures. Switching machinery to e-mobility and using hypoallergenic vegetation around the club grounds can help to reduce ozone and allergen exposure, respectively, to a certain degree.

Technical structural measures can also be used to reduce infection risks. For example, mosquito numbers can be curbed by avoiding having any areas of standing water (e.g., rain barrels, etc.) on site. On hot days, providing refrigeration for athletes’ food and ensuring a seamless cooling chain for event catering can help to prevent food-related infections.

### 3.2. Organisational Measures

The way that training and competitions are organised can also help to counteract the effects of climate change on health risks. Exposure to heat, UV radiation and ozone that is linked to the time of day and season can be counteracted by altering training and match times accordingly [17]. Milder winters allow for shorter winter breaks and longer summer breaks. Where athletes use school sports halls, the relevant caretaker should also be responsible for managing the temperature of these facilities during these periods. At events held on hot days, clubs can refrain from serving alcoholic beverages and, when serving coffee, switch to decaffeinated varieties in order to avoid putting additional strain on people’s cardiovascular systems (e.g., the spectators). In many sports, the official rules prohibit taking additional breaks or adequate textile UV protection (e.g., in football) [17]. Appropriate changes to the rules, as recently implemented in both football and beach volleyball could allow for more frequent player substitutions, additional breaks and more protective sports clothing (head coverings, design).

Pre-defined regulations should be established, not only concerning extreme heat but also regarding extreme weather events. In the event of an emergency (lightning, storm), referees, coaches and athletes can only act consistently and by the book once the rules governing interrupting, abandoning and scoring play have been clearly defined, as has been done in beach volleyball, for instance.

Organisational measures concerning protection from extreme weather events also include having adequate insurance coverage and regularly inspecting the premises so as to identify potential risks from falling branches, uneven ground or dead wood in good time. Such organisational measures can be extended to conduct a “climate risk audit” in which allergy triggers and infection risks can also be identified. This would include detecting wasp nests, oak processionary moths, possible breeding sites for mosquitoes and plants with allergenic potential (e.g., birch trees, ragweed or giant hogweed) at training grounds. When organising events, particularly on hot days, the risk of bacterial enteritis can be reduced by using hygiene checklists and choosing appropriate food and beverages.

### 3.3. Person-Related Measures

In addition, sports clubs and associations should also support the preventive action taken by individual athletes by implementing person-related measures. Responsible individuals such as coaches, referees and officials should pay special attention to particularly physically vulnerable groups such as children, senior citizens and athletes with pre-existing health conditions.

Within professional sport, training in hot weather can be made easier if the club provides pre-cooling methods (such as cooling vests or cold water immersion) [17,41]. At amateur level, coaches can offer water and cooling packs, and referees can instruct players to take additional hydration breaks in the shade or inside [17]. Designated cooling centres can be set up, showers can be opened at events, and cooler rooms can be made accessible during breaks in play. Additionally, catering can be expanded to include fruit and vegetables with a high water content.

With regard to extreme weather events such as storms and lightning strikes, clubs can provide coaches with information to raise their awareness, hand out prepared procedure plans and clearly assign responsibilities [17].

At major events, it may be advisable to hand out a protection package, comprising, e.g., a water bottle, sun protection, protective lip balm, a head covering, a simple pair of sunglasses and insect repellent to protect from wasps and mosquitoes, to each participant if necessary when they register and collect their starting numbers. Especially at full-day outdoor events, forgetting something such as sunscreen can result in severe UV damage if athletes are not offered alternatives on site. Person-related measures of this nature can thus address a number of the above-listed health risks associated with climate change. Sports clubs could finance these measures from donors or sponsors, for example.

In order to protect against the risks of tick-borne infections, relevant information boards could be put up in shower rooms as this is where players have the first opportunity to check their body for ticks after training. In water sports, infection risks can be reduced if coaches instruct athletes to tape up any wounds beforehand, to correctly store any food they may have brought with them and to completely refrain from training on open water if the water is known to be contaminated, e.g., with cyanobacteria.

### 3.4. Cross-Cutting Measures

Cross-cutting measures form the foundation of a comprehensive and successful prevention strategy. They can be effective at addressing multiple problems, thus consolidating the success of prevention efforts.

#### 3.4.1. Education and Training

The impact of climate change will be felt tangibly in some form by every sports club and institution. It therefore seems likely that a top-down approach would be helpful in keeping the various different stakeholders informed about climate change and its consequences. Curricula on the topic of sport and climate change should include information about the causes, health effects and possible prevention measures, delivered in a form that is appropriate for the respective target group. Relevant curricula can be effectively and efficiently adapted for different levels, settings and target groups.

Such a top-down approach includes national umbrella associations, ministries and universities as well as the sport-specific organisations from regional to local club level. In this context, suitable settings include symposiums and conferences, sport degrees and teacher training courses, education and training for coaches and athletes and sport association and club websites. Relevant target groups include association officials and political decision makers, sports science students and physical education teachers, coaches and trainers, professional and amateur athletes as well as parents and club managers who plan sports facilities and on-site events.

#### 3.4.2. Warning Systems and Heat Orientation Maps

Internet-based warning systems can warn users before and during training and competition about climate-induced health risks. Ideally, such applications allow location-specific searches. This system should integrate existing data sources such as heat warning systems, severe weather warnings, water level reports, UV indexes, ozone measurements, pollen count forecasts and endemic infection maps into one single app or presented on digital information boards on site. Germany’s National Meteorological Service has already launched a pilot project that includes several of the abovementioned aspects as well as providing cartographic illustrations, temporal forecasts and specific advice for at-risk groups [42].

In addition to this, at club level, we also propose producing local venue-specific heat orientation maps, where all measures for the prevention of heat-related health risks are set out in writing and plotted on a map. A venue-specific heat orientation map is a plan of the local facilities that depicts places such as shady areas, cool rooms, showers, protective shelters, water dispensers, bottle filling stations and sun protection stands (where protective clothing, sunscreen, etc., can be obtained) as well as defibrillators and first-aid stations (e.g., for dealing with cardiovascular complaints). This plan should be permanently installed at club premises (information boards, venue maps) and made easily accessible for every athlete (e.g., flyers, apps, etc.). This type of readily accessible and permanently available guidance should also include information about the multi-stage approach to be taken in the event of relevant thresholds being exceeded (e.g., outdoor temperature, ozone levels).

#### 3.4.3. Cooperation and Coordination

The complexity of the health-related impacts of climate change means that interdisciplinary and transdisciplinary cooperation is recommended between scientific experts from a range of specialist fields and stakeholders active in the world of sport. From a medical perspective, a prevention plan should include medical expertise from various specialists, e.g., sport medicine, internal medicine, dermatology, toxicology, allergology, infectiology and pulmonary medicine. Furthermore, other experts such as climate scientists, sports scientists, psychologists, educators and biologists as well as landscape gardeners, urban planners, architects and insurance experts (reinsurers and natural hazard insurers) should also contribute their expertise. Finally, it is essential to include the practical experience of active athletes, coaches and club officials from both elite and mass sport. In this context, national umbrella associations could take on a coordinating role in the development, implementation and communication of a scalable prevention plan for their respective sport. To this end, a centralised website hosted by the central association would be a suitable place to efficiently and cost-effectively provide such information to member associations, athletes and other interested parties. The typical hierarchical structure of sport associations opens up synergies, for example, through content developed centrally by the umbrella associations, which can then be adapted for—or at least linked to by—downstream associations and clubs.

#### 3.4.4. Evaluation

As with any public health intervention, all measures aimed at protecting against the impact of climate change should be evaluated [43]. Only in this way can successes and failures be objectified, improvements introduced and developments depicted. To this end, the first steps are to define relevant outcome parameters for operationalisation, to develop an indicator system and, finally, to carry out a standardised evaluation process. Such a system should include baseline and follow-up values for target parameters such as room and surface temperatures, measurement data for UV, ozone and pollen levels and illness numbers and data on the costs, acceptance and reach of the measures. In terms of methodology, it is recommended to adopt a multi-modal approach that comprises classic measurement data, audits and surveys. Lastly, using modern monitoring systems, it is possible to link local, club-level measures with official climate data.

## 4. Discussion

### 4.1. Athletes as a Vulnerable Population Subgroup

When viewed in the context of the entire history of the Earth, global climate change is progressing at breakneck speed. From an individual point of view, initial indications are just beginning to emerge showing the specific impact of climate change on sports. The potential to perceive climate change as “a catastrophe in slow motion” might explain why little sports science research has been conducted to date on this global challenge. This article picks up on calls for coordinated prevention programmes aimed at protecting athletes and describes what we believe to be one of the central fields of research, intervention and prevention for the future [3,44].

From other fields of prevention work such as tobacco, alcohol, obesity or drug prevention, it is well-established that behavioural prevention measures—which affect the athletes themselves—are always particularly efficient and effective when they are accompanied by prevention measures, which improve structures. The SC^3^ model thus follows a structural approach to prevention work. This approach is both ethical and efficient—ethical because sports clubs and associations are obliged to protect their athletes and efficient because meso-level measures are a long-lasting, cost-efficient way to reach a large number of affected people [3]. Tools are available from the field of sport management that can be used to define the specific vulnerability of a certain club—and not the active athletes in the club—to climate change [45].

### 4.2. Limitations of the Model

There are several limits to the model presented here. The first aspect is that we mainly address those climatic changes that are typically expected for high-income countries in the mid-latitudes. First, it should not be forgotten that low- and middle-income countries are, of course, also affected by the consequences of climate change. Specifically, it can be assumed that the climate impacts there will often be even more severe, the vulnerability higher and the available resources lower [39,46,47,48]. Last but not least, low- and middle-income countries are often located closer to the equator. Such regions are even more affected by climatic changes because of their geographical location. Recent publications emphasise that for mid-latitudes, global warming means milder winters and thus more favourable conditions for outdoor activities, while for many subtropical and tropical regions, climate change will create even hotter and thus less favourable conditions for outdoor sports and physical activity [46,47]. Thus, the model proposed here should be further adjusted for low- and middle-income countries in the future.

Another aspect is an adequate evaluation. Thus, each individual measure—of which only examples are given here—would have to be assessed and evaluated in terms of its effectiveness and efficiency against the background of regional or local conditions. For example, the Building Resilience Against Climate Effects (BRACE) framework describes in detail how this can be done [39].

## 5. Conclusions

Climate change mitigation covers all measures that are appropriate for stopping, delaying or reducing climate change [2]. Mitigation is thus an upstream approach [1]. In contrast, climate change adaptation refers to making adjustments to accommodate the manifold consequences of climate change [45]. Adaptation therefore represents a downstream approach.

The fact that the proposal presented here deals solely with adaptation measures does not mean that sports organisations should not also commit to mitigation strategies as well as adaptation measures. On the contrary, the relationship between climate change and sport is bidirectional [45]. Sport not only suffers from but also causes greenhouse gas emissions and thus climate change [13,49]. Major events such as international and national tournaments, championships and the league system at all levels cause considerable amounts of sport-related mobility that affects CO_2_ levels [50]. Sport also impinges on attractive natural environments such as coastal areas, forests and mountains. Sports facilities require energy, e.g., for air conditioning and maintenance. Sports clubs sell products and generate waste (e.g., sports gear, equipment, technology, paper, catering). There is therefore considerable potential to save energy and raw materials within the typical activities related to sports enterprises [3]. In short: sport can play an important role in climate action worldwide [13]. Whereas this article presents a suitable adaptation strategy, the United Nations has published a mitigation strategy, the UNFCCC “Sports for Climate Action” framework [51]. Sport is responsible for both.

## Figures and Tables

**Table 1:** Health risks linked to climate change and exemplary intervention measures at organisational level.

Climate-Related Health Risks	Proposed Intervention Measures		
	Technical Measures	Organisational Measures	Person-Related Measures
Increased thermal stress	modern insulation technology and glazing energetic renovation avoidance of heat-storing building materials green roofs and facades climate-resistant planting adaptation of lawn care, watering and pruning natural and artificial shading installation of water dispensers	time and/or place relocation of training, competitions and training camps (also after indoor) changeover to evening floodlit competitions ventilation of training and shelter areas acclimation-oriented arrival and event planning discontinuation of the sale of alcoholic and caffeinated beverages adjustment of rules and regulations (thresholds that allow more frequent player changes, shortening of game duration, additional breaks)	provision of pre-cooling methods designation of cooling centres and opening of shower rooms use of water sprinklers distribution of drinking bottles intermittent breaks with common pulse checks additional common drinking breaks with quantity recommendation adjustment of training schedules and reduction of training volume nutritional advice provision of pulse oximeters resp. peak flow meters extension of catering to include water-rich fruits and vegetables (melons, cucumbers, tomatoes, strawberries, peaches)
Extreme weather events	construction and designation of lightning shelters/rooms flood protection, e.g., by rainwater retention installation of backwater protection efficient use of water and closed water cycle as far as possible	regulations for the interruption of play scheduled and regular inspection of training areas/grounds (broken branches, dead wood, terrain damage, etc.) flexibility in planning and event postponement in case of early weather warnings closure of training areas, ski areas and hiking tours	information on and handing out of plans for specific weather events (emergency plans) regulation of the role assignments of the responsible persons
Increased UV exposure	natural (trees) and artificial shading (awnings) albedo-friendly ground coverings (lawns instead of sealing) roofing of break, spectator and referee areas	shortening of the winter break and extension of the summer break scheduling sufficient breaks as early as the schedule preparation stage relocation of breaks to shaded areas setting up mobile sunshades and dispensers for sunscreen products	distribution of sunscreen and lip protection (e.g., with the start number) education by “UV pilots” (responsible persons) on site free distribution, rental or sale of sunscreens, headgear, sunglasses, UV-safe clothing to athletes and spectators at events
Increased air pollution	conversion of the machine park to electric mobility abandonment of exhaust-intensive devices and machines	shifting training time to early morning and late evening hours (before 11:00, after 18:00). shortening of running distances and times reduction of air pollution through off-track routing and widespread closure to traffic training interruption (e.g., at 1 h ozone value > 150 μg/m^2^)	warning/training change, e.g., at 1 h ozone value > 120 μg/m^2^ regular symptom query during training and competition
Increased exposure to allergens	hypoallergenic greening and tree species (avoidance of birch and olive trees, etc.)	walk-throughs and audits to detect wild allergenic plants, oak process moth, wasp nests, water areas (such as rain barrels)	distribution of wasp repellents to competitors, officials and spectators
Spread of infectious diseases	reduction of standing freshwater surfaces (puddles, rain barrels) cold chains/possibility for provisions and catering	temperature-adapted catering hygiene checklists for events and competitions regular water sampling including publicly available analysis reports	information boards in the showers (e.g., on ticks). refrain from open water or at least from kayak roll training when exposed to cyanobacteria and other pathogens installation of disinfectant dispensers for surfaces and hands common hand disinfection (e.g., before breaks) stopping training or taping off in case of wounds provision of water disinfection and filtration (e.g., in mountain sports) instructions for airtight sealing of provisions, waste and drinks

## Data Availability

Not applicable.
